# Validation of *PIN 3* physical activity survey in low-income overweight and obese young mothers

**DOI:** 10.1186/s12889-015-1493-z

**Published:** 2015-02-10

**Authors:** Mei-Wei Chang, Derek Hales, Roger Brown, Dianne Ward, Ken Resnicow, Susan Nitzke

**Affiliations:** Michigan State University, College of Nursing, 1355 Bogue Street, RM C346, East Lansing, MI 48824 USA; University of North Carolina-Chapel Hill, School of Public Health, Gillings School of Global Public Health, Chapel Hill, NC USA; University of Wisconsin-Madison, School of Nursing, 600 Highland Avenue, Madison, WI 53792 USA; University of Michigan, School of Public Health, 1415 Washington Heights, Ann Arbor, Michigan 48104 USA; Department of Nutritional Sciences, University of Wisconsin-Madison, 1415 Linden Drive, Madison, WI 53706 USA

**Keywords:** Validity, Physical activity survey, Low-income women

## Abstract

**Background:**

Existing physical activity surveys have not been validated for use with low-income overweight and obese young mothers. This study aimed to validate the Pregnancy Infection and Nutrition 3 (PIN3) physical activity survey and to explore whether its validity varied by race/ethnicity and body mass index (BMI) category when including or excluding child and adult care activities in the target population.

**Methods:**

Participants were recruited from the Special Supplemental Nutrition Program for Women, Infants, and Children (WIC) and were asked to fill out the PIN3 survey and wear an Actigraph accelerometer. Validity was assessed (N = 42) using Spearman correlation coefficient.

**Results:**

Regardless of inclusion or exclusion of child and adult care activity, the PIN3 survey showed evidence of validity for moderate (correlation coefficients 0.33 [p = 0.03]; 0.40 [p = 0.08]) but not vigorous (−0.01 [p = 0.91]; −0.06 [p = 0.69]) physical activity. The mean minutes per week spent in moderate, vigorous and moderate-vigorous physical activity measured by the PIN3 were substantially higher than when measured by accelerometer, for example, 588 (PIN3) versus 148 (accelerometer) minutes per week. Also, correlations between self-reported and objective monitored activity varied substantially by race/ethnicity and BMI category, for example, 0.29 (p = 0.18) for overweight women versus 0.57 (p = 0.007) for obese women; 0.27 (p = 0.20) for African American versus 0.66 (p = 0.001) for white.

**Conclusions:**

The PIN3 survey may be adequate for many applications where quick and practical assessments are needed for moderate physical activity data in low-income overweight and obese young mothers. The substantial differences in mean minutes per week between the PIN3 and accelerometer may be due to over-reported physical activity by the study participants.

**Trial registration:**

Clinical Trials Number: NCT01839708

## Background

Nearly 60% of American women 20–39 years old are overweight (27%) or obese (32%) [1]. Overweight and obesity increase individuals’ risk of type 2 diabetes and cardiovascular disease (CVD) [2-4]. Compared to normal weight women, overweight and obese women are less likely to engage in physical activity [5]. Also, low-income women are less likely to be physically active than high-income women [6]. Sedentary lifestyle increases risk of weight gain [7,8], insulin resistance [8] and obesity [9] and is associated with cardiovascular and cancer mortality [10]. On the other hand, regular physical activity prevents several chronic diseases (for example, diabetes, CVD, hypertension, obesity, and depression) and premature death [11]. Physical activity guidelines have been developed for adults to achieve health benefits [12]. Thus, practical assessment tools are needed to allow users to monitor their progress and evaluate the effectiveness of interventions designed to promote physical activity. While objective measurements (for example, Actigraph accelerometer) provide more accurate physical activity measurement than self-reported surveys, it may not be feasible for most epidemiology and intervention studies to collect physical activity data via direct measurement.

The Pregnancy Infection and Nutrition 3 (PIN3) survey assesses several types of self-reported physical activity and measures perceived intensity levels that are not addressed by other surveys (e.g., Kaiser Physical Activity [13] and International Physical Activity Questionnaires [14]). The PIN3 was originally developed using a sample of pregnant women who were predominately White with college educations [15].

Women spend a significant amount of time each day in activities for household, child care and work [16,17]. There is evidence that young mothers of young children have difficulties categorizing activities related to child care and household tasks and find accurately recalling frequency and duration challenging [17].

A previous study that validated a *RESIDE* physical activity questionnaire in low-income middle aged, overweight and obese women found similar correlations (validity) between non-Hispanic blacks and other participants [18]. Other studies have reported reduced validity of physical activity surveys among individuals with higher body mass index (BMI) [18-20]. However, whether validity evidence varies by race/ethnicity and BMI category in low-income overweight and obese young mothers remains unknown. Also, no physical activity survey has been validated for the use of low-income overweight and obese young mothers. Therefore, the purpose of this study was to validate the PIN3 physical activity survey and to explore whether its validity varied by race/ethnicity and BMI category when including or excluding child and adult care activities in low-income overweight and obese mothers aged 18 to 39 years.

## Methods

### Study design and sample

This study was conducted as an auxiliary study of a large intervention study called *Mothers In Motion* that is aimed to help low-income overweight and obese young mothers prevent weight gain via promoting stress management, healthy eating, and physical activity [21]. Participants were recruited from the Special Supplemental Nutrition Program for Women, Infants, and Children (WIC). To be eligible for WIC, individuals must have an annual household income at or below 185% of the federal poverty line. WIC provides nutrition consultation and other services to pregnant, postpartum, and breastfeeding women and children under 5 years old.

Prior to data collection, we conducted cognitive interviews with the target audience (3 African American and 2 White) to assess their understanding of the PIN3 survey instructions and questions. In general, these women had difficulties in understanding the instructions and some terminology such as ‘mowing’ and ‘lifting children’. Thus, we worked closely with these women to revise the instructions to improve the clarity. We also modified the wording (for example, ‘mowing’ was modified to ‘push a lawn mower’) and we removed questions about lifting children or adults.

Participants were recruited from April through September 2013 via personal invitation while women were waiting for their WIC appointments at collaborating WIC offices in Michigan. To be eligible, participants were required to be non pregnant African American or non-Hispanic White, 18–39 years old, at least 6 weeks postpartum, and able to walk more than 1 block without resting. They also understood and spoke English, had no self-reported type 1 or 2 diabetes and had measured BMIs between 25.0 and 39.9 kg/m^2^. Height was measured without shoes to the nearest 0.1 cm using a wall-mounted stadiometer. We measured body weight to the nearest 0.2 pounds on an electronic digital scale (Seca 869, Germany) with the participants wearing light clothing and no shoes.

Eligible participants were asked to complete a self-administered PIN3 physical activity survey at the WIC waiting room area. Then, our research assistants provided verbal and written instructions with a picture on how to wear the Actigraph accelerometer on the right hip under clothing. They also helped the participants put on the accelerometer using an adjustable elastic belt and instructed them maintain their usual level of regular physical activity while wearing it. Then, participants demonstrated how to put on the accelerometer before leaving the WIC office. They were asked to wear the monitor for 10 or more waking hours during 7 consecutive days. They removed the monitor when swimming, bathing/showering, and sleeping at night. Participants completed a daily log to record when the accelerometer was worn. The research assistants called participants every other day to remind them to wear their accelerometer. After 7 days, participants mailed the accelerometer to the study office using a pre-stamped envelope. Participants read and signed a written consent form approved by the Institutional Review Board of Michigan State University and Michigan Department of Community Health.

### Measurements

#### Physical activity survey

The PIN3 survey (24 items) measures frequency, duration, and perceived intensity of physical activity that is done for recreation activity (4 items), indoor (5 items) and outdoor (4 items) household tasks, child and adult care activity (5 items), transportation activity (2 items) and activity at work and school (4 items). The survey has established psychometric properties (correlations) validated by accelerometers and assessed using Spearmen correlation [15]. Coefficients ranged from 0.12 to 0.23 for perceived intensity (hours/week) and from 0.28 to 0.34 for the absolute intensity (metabolic equivalent [MET]). The test retest reliability for moderate (MPA), vigorous (VPA) and moderate-vigorous (MVPA) physical activity ranged from 0.40 to 0.56 for the perceived intensity and ranged from 0.73 to 0.82 for the absolute intensity.

Using recreational activity as an example, participants were first prompted by questions related to recreational activity or exercise that caused at least some increase in breathing or heart rate. They responded to questions such as “In the past 7 days, did you participate in walking for exercise?” If they responded “Yes”, then they provided frequency (Fill in the blank: how many times did you do the activity?), duration (Fill in the blank: on average, how many minutes or hours did you do the activity each time?) and perceived intensity level (fairly easy, somewhat hard, hard, or very hard [in a multiple choice formatting]). These questions were repeated for indoor and outdoor household tasks, child and adult care activity, transportation activity and activity at work and school [15].

### ActiGraph accelerometer

The ActiGraph GT1M (Pensacola Florida, USA) is a small uniaxial accelerometer which is a valid and reliable tool to quantify physical activity in adults [22,23]. The accelerometer is designed to detect vertical acceleration from normal human movements and disregard high frequency movements such as vibrations. The accelerometer provides detailed information about the frequency, duration, and intensity of physical activity. In the current study, the GTIM accelerometer was set to record activity counts at 1- min epoch. We applied non-wear definitions and outcome cut points that were used in the National Health and Nutrition Examination Survey [24] and used SAS to clean non-wear times and to compute outcomes. Non-wear time was defined as an interval of at least 60 consecutive minutes of zero physical activity counts [24]. Three primary outcomes were estimated: minutes of MPA (counts per minute 2020–5998), minutes of VPA (counts per minute 5999+), and minutes of MVPA (counts per minute 2020+). Summary outcomes were computed if a woman had at least 3 days and at least 7 hours per day of physical activity count data.

### Statistical analysis

We defined MPA as 3 to 5.9 METs and VPA at or greater than 6.0 METs. All analyses were performed using Stata 13.1 [25]. We performed descriptive analysis and calculated means and standard deviations for minutes of MPA, VPA and MVPA per week using data from the PIN3 survey and accelerometer. Validity was assessed using Spearman rank correlation to compare results of accelerometer to the modified PIN3 survey. Below, we describe the calculation of minutes and METs per week for both PIN3 and accelerometer.

Total minutes/per week

PIN3: frequency × duration (in minutes)

Accelerometer: minutes (total minutes/days of wearing) × 7 days

Total METs/per week

PIN3: frequency × duration (in minutes) × perceived intensity.

Using walking as an example:

6 times (frequency) × 50 minutes (duration/each time) × fairly easy (perceived intensity; coded as light activity and used 2.5 METs from the compendium of physical activity [26,27]).

Accelerometer: minutes (total minutes/days of wearing) × 7 days × 3 METs (light intensity)

## Results

A total of 56 participants completed the PIN3 survey. However, only 42 data sets were included in our analyses because they contained self-reported PIN3 survey and suitable accelerometer wearing time (at least 3 days and at least 7 hours per day). Data from 14 women were excluded because 4 did not return the accelerometer, 4 wore the monitor less than 3 days, and 6 had technical instrument failures. Table 1 presents demographic characteristics of 42 women by race and BMI category. Most participants were African American or overweight.

**Table 1:** **Demographic characteristics**

	**African American (n = 23)**	**White (n = 19)**	**Overweight (n =22 )**	**Obese (n = 20)**	**Total (N = 42)**
	**M (SD)**	**M (SD)**	**M (SD)**	**M (SD)**	**M (SD)**
**Age (years)**	27.2 (4.98)	30.0 (4.48)	28.4 (4.46)	28.6 (5.48)	28.5 (4.91)
**Postpartum status (years)**	1.56 (1.15)	1.98 (1.35)	1.68 (1.41)	1.85 (1.07)	1.76 (1.25)
**Body mass index (kg/m** ^**2**^ **)**	32.5 (5.70)	29.1 (2.61)	27.5 (1.69)	34.6 (4.42)	30.9 (4.84)
	**n (%)**	**n (%)**	**n (%)**	**n (%)**	**n (%)**
**Smoking status**					
Never smoked	11 (47.8%)	7 (36.8%)	9 (40.9%)	9 (45.0%)	18 (42.8%)
Smoked, but quit	5 (21.7%)	5 (26.3%)	7 (31.8%)	3 (15.0%)	10 (23.8%)
Smoker	7 (30.4%)	7 (36.8%)	6 (27.2%)	8 (40.0%)	14 (33.3%)
**Education**					
8^th^ grade or less	1 (4.3%)	0 (0.0%)	1 (4.5%)	0 (0.0%)	1 (2.3%)
Some high school	4 (17.3%)	2 (10.5%)	3 (13.6%)	3 (15.0%)	6 (14.2%)
High school graduate	6 (26.0%)	4 (21.0%)	4 (18.1%)	6 (30.0%)	10 (23.8%)
Some college or technical school	11 (47.8%)	10 (52.6%)	11 (50.0%)	10 (50.0%)	21 (50.0%)
College graduate or higher	1 (4.3%)	3 (15.7%)	3 (13.6%)	1 (5.0%)	4 (9.5%)
	**n (%)**	**n (%)**	**n (%)**	**n (%)**	**n (%)**
**Employment status**					
Full time	4 (17.3%)	3 (15.5%)	3 (13.6%)	4 (20.0%)	7 (16.6%)
Part time	4 (17.3%)	3 (15.5%)	4 (18.1%)	3 (15.0%)	7 (16.6%)
Unemployed	10 (43.4%)	3 (15.5%)	6 (27.2%)	7 (35.0%)	13 (30.9%)
Homemaker	2 (8.7%)	4 (21.0%)	4 (18.1%)	2 (10.0%)	6 (14.2%)
Self-employed	1 (4.3%)	4 (21.0%)	3 (13.6%)	2 (10.0%)	5 (11.9%)
Student	2 (8.7%	1 (5.2%)	1 (4.5%)	2 (10.0%)	3 (7.1%)
Other	0 (0.0%)	1 (5.2%)	1 (4.5%)	0 (0.0%)	1 (2.3%)
**Depression**					
No	18 (78.3%)	12 (63.2%)	16 (72.7%)	14 (70.0%)	30 (71.4%)
Yes	5 (21.7%)	7 (36.8%)	6 (27.3%)	6 (30.0%)	12 (28.6%)
**Currently breastfeeding**					
No	20 (86.9%)	15 (78.9%)	17 (77.2%)	18 (90.0%)	35 (83.3%)
Yes	3 (13.1%)	4 (21.2%)	5 (22.8%)	2 (10.0%)	7 (16.7%)

Overall, when including child and adult care activity measured by the PIN3 (Table 2), the mean minutes per week spent in MPA, VPA, and MVPA measured by PIN3 were substantially higher than measured by the accelerometer. For example, 588 (PIN3) versus 148 minutes per week (accelerometer) for all participants; 448 (PIN3) versus 152 (accelerometer) minutes per week for African American; 758 (PIN3) versus 144 (accelerometer) minutes per week for White; 352 (PIN3) versus 142 (accelerometer) minutes per week for overweight; 847 (PIN3) versus 156 (accelerometer) minutes per week for obesity. However, when excluding child and adult care activity (Table 2), the mean count minutes per week spent in MPA measured by PIN3 were either higher or lower than measured by the accelerometer: 234 (PIN3) versus 148 (accelerometer) minutes per week for all participants; 246 (PIN3) versus 152 (accelerometer) minutes per week for African American; 221 (PIN3) versus 144 (accelerometer) minutes per week for White; 80 (PIN3) versus 142 (accelerometer) minutes per week for overweight; 405 (PIN3) versus 156 (accelerometer) minutes per week for obesity.

**Table 2:** Mean and standard deviation values* for the ActiGraph accelerometer and PIN 3 physical activity survey**

	**Actigraph accelerometer**			**PIN3 survey including child and adult care activity** ^**1**^			**PIN3 survey excluding child and adult care activity** ^**2**^		
	**MPA**	**VPA**	**MVPA**	**MPA**	**VPA**	**MVPA**	**MPA**	**VPA**	**MVPA**
	**M (SD)**	**M (SD)**	**M (SD)**	**M (SD)**	**M (SD)**	**M (SD)**	**M (SD)**	**M (SD)**	**M (SD)**
**All participants**	148.2 (91.7)	2.0 (3.9)	150.2 (93.1)	587.9 (1278.3)	52.6 (261.5)	640.5 (1406.0)	234.4 (788.3)	9.2 (43.1)	243.6 (792.6)
**Race**									
African American	151.6 (94.1)	3.1 (4.5)	154.8 (96.6)	447.5 (1076.9)	0	447.5 (1076.9)	245.8 (1003.1)	0.0 (0.0)	245.8 (1003.1)
White	144.0 (91.1)	0.7 (1.6)	144.7 (91.1)	757.8 (1499.7)	116.3 (384.7)	874.2 (1726.2)	220.5 (430.3)	20.5 (63.2)	241.0 (448.3)
**Body mass index (BMI)**									
Overweight: 25.0-29.9 kg/m^2^	141.5 (94.7)	2.4 (4.8)	144.0 (97.2)	352.2 (563.3)	13.1 (42.6)	365.4 (570.3)	79.7 (255.6)	6.8 (31.9)	86.5 (261.6)
Obesity: 30.0-39.9 kg/m^2^	155.5 (90.1)	1.5 (1.8)	157.0 (90.5)	847.1 (1743.7)	96.0 (376.6)	943.1 (1929.3)	404.5 (1100.2)	12.0 (53.6)	416.5 (1104.5)

^*^Minutes per week. ^**^N = 42: 23 African American, 19 White =19; 22 overweight, 20 obese.

^1^Sum of all activities: recreational activity, indoor and outdoor household task, child and adult care activity, transportation activity, activity at work and school. ^2^Sum of all activities excluding child and adult care activity. MPA = moderate physical activity, VPA = vigorous physical activity. MVPA = moderate-vigorous physical activity.

The Spearmen correlations between the PIN3 survey and accelerometer measures were 0.40 for MPA and 0.39 for MVPA when including child and adult care activity (Table 3). The correlations decreased from 0.40 to 0.33 for MPA and from 0.39 to 0.32 for MVPA when excluding child and adult care activity (Table 4). There were no correlations between the PIN3 survey and the accelerometer data for VPA regardless of the inclusion or exclusion of child and adult care activity.

**Table 3:** Correlation between ActiGraph and self-reported PIN3 physical activity survey including child and adult care activity* ^**1**^

	**ActiGraph accelerometer vs. PIN3 physical activity survey**		
	**MPA**	**VPA**	**MVPA**
**All participants**	0.40 (p = 0.008)	−0.01 (p = 0.91)	0.39 (p = 0.01)
**Race**			
African American	0.27 (p = 0.20)	0.00 (p = 1.00)	0.26 (p = 0.21)
White	0.66 (p = 0.001)	0.37 (p = 0.11)	0.65 (p = 0.002)
**Body mass index (BMI)**			
Overweight: 25.0-29.9 kg/m^2^	0.29 (p = 0.18)	0.25 (p = 0.25)	0.27 (p = 0.21)
Obesity: 30.0-39.9 kg/m^2^	0.57 (p = 0.007)	−0.25 (p = 0.26)	0.57 (p = 0.007)

^*^N = 42. ^1^Sum of all activities: recreational activity, indoor and outdoor household task, child and adult care activity, transportation activity, activity at work and school. MPA = moderate physical activity, VPA = vigorous physical activity. MVPA = moderate-vigorous physical activity.

**Table 4:** Correlation between ActiGraph and self-reported PIN3 physical activity survey without child and adult care activity* ^**1**^

	**ActiGraph accelerometer vs. PIN3 physical activity survey**		
	**MPA**	**VPA**	**MVPA**
**Total** ^**1**^	0.33 (p = 0.03)	−0.06 (p = 0.69)	0.32 (p = 0.03)
**Race**			
**African American**	0.44 (p = 0.03)	0.00 (p = 1.00)	0.44 (p = 0.03)
**White**	0.41 (p = 0.07)	0.11 (p = 0.64)	0.41 (p = 0.08)
**Body mass index (BMI)**			
Overweight: 25.0-29.9 kg/m^2^	0.18 (p = 0.40)	0.17 (p = 0.43)	0.15 (p = 0.48)
Obesity: 30.0-39.9 kg/m^2^	0.51 (p = 0.02)	−0.26 (p = 0.25)	0.52 (p = 0.01)

^*^N = 42. ^1^Sum of all activities: recreational activity, indoor and outdoor household task, transportation activity, activity at work and school. MPA = moderate physical activity, VPA = vigorous physical activity. MVPA = moderate-vigorous physical activity.

The correlations differed substantially by race/ethnicity and BMI category for MPA and MVPA (Tables 3 and 4). When including child and adult care activity in the analysis (Table 3), the correlations for White (0.66) and obese (0.57) women were stronger than African American (0.27) and overweight (0.29) women for the MPA. When child and adult care activity was removed from the analysis (Table 4), the correlation for African American (0.44) and obese (0.51) women were stronger than White (0.41) and overweight (0.18) women for the MPA. Regardless of race/ethnicity, BMI category, and inclusion or exclusion of child and adult care activity, no correlations were found for VPA.

## Discussion

This study evaluated the validity of a physical activity survey for use among low-income overweight and obese young mothers. Regardless of the inclusion or exclusion of child and adult care activity, the correlations between the PIN3 and accelerometer were moderate (0.32-0.40) for MPA and MVPA for the total participants; thus indicating that the PIN3 may be an acceptable measure for MPA but not VPA among the target audience. Systematic reviews assessing psychometric properties of self-reported physical activity measures in adults showed a mean (such as, 0.22 for MPA, 0.32 for VPA [28], or 0.37 [regardless of intensity]) [29] or median correlations (such as, 0.3) that were less than 0.40 [30]. The differing patterns of correlations between self-reported surveys and the objective measures might have been due to the type of objective measurement used (for example, accelerometer, doubly labeled water, or pedometer) [28].

We identified substantial differences in validity evidence by race/ethnicity and BMI category which are inconsistent with prior research. A recent study that evaluated the validity of a physical activity survey in middle aged, low-income overweight and obese women found that the correlation between the physical activity survey and accelerometer measured physical activity was stronger for women with lower BMIs (≤35.0) than women with higher BMIs (>35.0) [18]. Also, these researchers found similar correlations between non-Hispanic blacks and others [18]. The variations in findings may have resulted from differences in surveys, demographic characteristics, and/or BMI cut off levels. The validity evidence showing a stronger relationship between physical activity and obese compared to overweight status in women was most likely due to a small sample size.

A systematic review showed that on average women over-reported their physical activity levels by 138% higher when using self-report than when physical activity was measured by accelerometers [28]. Also, overweight and obese women were more likely to over-report physical activity than normal weight women [31,32]. Over reporting of physical activity as high as 167-370% has been reported when compared to physical activity measured by accelerometer [18,33], which may be due to inaccuracy of participants’ recall data. In our ongoing *Mothers In Motion* study [21], some participants self-reported the presence of a learning disability and about 40% of our participants had a high school or less education. Recalling duration and frequency of physical activity requires numeracy skills and may have been challenging for these women. Thus, some of our participants’ data might have been affected by difficulties in quantifying the frequency, duration, and intensity of physical activity in the past week. Also, they might have misinterpreted survey instructions, reporting periods, or activity categories [17] due to limitations in prose/reading literacy. We carefully revised the survey instructions prior to data collection to minimize misinterpretation of survey instructions but we could not eliminate the challenges encountered with very low levels of numeracy or reading abilities. We have also learned that additional improvements may be needed in the formatting of the activity categories to accommodate literacy limitations.

The PIN3 survey data were collected prior to the collection of accelerometer data. It is possible that one or both data collection periods were not typical for the study participants. Previous researchers have found that when inclusion of level of perceived intensity was included, participants tended to report higher level of intensity [28]. A prior focus group study of young mothers with young children found that some participants misinterpreted the reference period and units of measurement (e.g., reporting hours/month instead of hours/day) [17]. Women also indicated difficulties in recalling last week’s physical activity accurately because it might not have been their typical week [17].

Social desirability may play a role in over-reporting physical activity [34]. Moreover, accelerometers have been reported to underestimate physical activity [35]. The accelerometer detects activities involved in the vertical plane from the hip where the monitor is worn but does not record upper body activities (For example, upper body resistance training or carrying a child when standing) [35]. Our participants were mothers of young children and about 48% were obese. Therefore, it is possible that the accelerometer placement was tilted and not in the vertical position to accurately count steps when worn on the waist of these obese participants and/or when they carried young children.

We found that low-income overweight and obese young mothers over-reported their physical activity substantially when child and adult care activity was included in analysis. Our participants were young busy mothers with young children; they might have double counted some same child and adult care activity [17,36]. Also, they might have included multiple activities for the same period of time due to multi-tasking, e.g., childcare and housework [17].

We only included data from women who wore their accelerometer at least 3 days and at least 7 hours per day. Therefore, the inclusion of the accelerometer data for less than 7 days may not have provided a reliable estimate of habitual physical activity [37].

Limitations to this study include the small sample size, thus we were unable to detect important relationships in subgroups, such as race/ethnicity and BMI category. Although simultaneous data collection via two methods would have been ideal, we collected PIN3 survey data prior to asking the participants to wear the accelerometer. This was done to maximize efficiency of data collection of the survey. We have extensive experience working with the target audience and learned that collecting data while the participants waiting for their appointments at WIC offices is much easier than collecting data via phone. Our study participants experience stressful and unstable life situations. We made phone reminders 3 times a week to remind participants to wear the accelerometer but in most cases we were unable to contact the study participants directly, often because phone services had been temporarily or permanently disconnected. In addition to disconnected phones, changing phone numbers and limited cell phone minutes made it difficult to reach many participants, especially those living in the unsafe neighborhoods [38]. When we were able to reach the participants; data collection often was interrupted by elements within their stressful and unstable life situations, such as frequent demands for attention from young children [17]. Another limitation is that data were collected from 3 geographic locations in Michigan and about two-third participants were recruited from a WIC office serving 80% of enrollees receiving Medicaid. Therefore, our sample may not be representative of all WIC enrollees in US. Finally, instead of using 7 days with at least 10 hours of record data per day, we included data from at least 3 days and at least 7 hours per day.

## Conclusion

In conclusion, our results showed that the PIN3 survey overestimated physical activity, especially when including child and adult care activity. The PIN3 survey may be used to measure MPA but not VPA among low-income overweight and obese young mothers. Use of the PIN3 survey to gather information about child and adult care physical activity needs to be used with caution. Further studies with a larger sample size may be needed to identify effective ways to collect child and adult care activity and to validate the PIN3 in specific population groups.

## Abbreviations

BMI: Body mass index
CVD: Cardiovascular disease
MET: Metabolic equivalent
MPA: Moderate physical activity
MVPA: Moderate-vigorous physical activity
PIN3: Pregnancy Infection and Nutrition 3
VPA: Vigorous physical activity
WIC: The Special Supplemental Nutrition Program for Women, Infants, and Children
